# Draft genome sequence of NDM-5, CMY-42 carrying carbapenem-resistant *Escherichia coli*

**DOI:** 10.1128/mra.00638-23

**Published:** 2023-11-30

**Authors:** Yogesh Mudaliar, Santha Kalaikumari S., Nithyasri M., Mathi Roshini N. B., Marquess Raj, Meera J., Elavarashi Elangovan, Kumar Perumal

**Affiliations:** 1 Department of Biotechnology, Sri Ramachandra Institute of Higher Education and Research, Chennai, Tamil Nadu, India; 2 Department of Biotechnology, Hindustan College of Arts & Science, Chennai, Tamil Nadu, India; 3 Apollo Diagnostics, Regional Reference Laboratory, Chennai, Tamil Nadu, India; University of Maryland School of Medicine, Maryland, USA

**Keywords:** CREC-1, ST2083, *Escherichia coli*, NDM-5, CMY-42, carbapenem resistant

## Abstract

We report the draft genome sequence of carbapenem-resistant *Escherichia coli* (CREC-1) of sequence type (ST2083) isolated from a urine sample of a 2-year-old female toddler carrying antimicrobial resistance genes *blaNDM-5* and *blaCMY-42* which displays resistance against multiple classes of antibiotics notably β-lactam antibiotics, cephalosporins, and carbapenem and codes for several virulence factors.

## ANNOUNCEMENT

Urinary tract infection increased from 252.25 million in 1990 to 404.61 million in 2019 globally (1). There has been an increase in concern over *Escherichia coli*-related urinary tract infections, particularly those caused by carbapenem resistance in India (2). The study was approved by the Institutional Ethics Committee for student projects at Sri Ramachandra Institute of Higher Education and Research (Approval number-CSP/21/MAR/92/265) for the clinical organism used in the study. Carbapenem-resistant *Escherichia coli* (CREC-1) was obtained from a urine sample of a 2 years, 2 months, and 20 days old female toddler, from a diagnostic laboratory in Chennai, India. The strain was cultured in MacConkey agar and incubated at 37°C for 24 h and biochemically characterized as *E. coli* by IMViC and MALDI-TOF MS (3). Antimicrobial susceptibility testing against various antibiotics was performed by disc diffusion assay from a single colony of strain according to Clinical and Laboratory Institute guidelines (4). Genomic DNA was extracted from overnight nutrient broth culture of *E. coli* maintained at 37℃ by using sodium dodecyl sulfate method (5). The library preparation was carried out using NEBNext Ultra DNA Library Prep Kit. The whole-genome sequencing was performed using the Illumina NovaSeq 6000. There were 5,597,164 paired raw reads (R1 and R2) generated, with a mean length of 150 bp. The Illumina adapter sequences are removed from FASTQ files, and AdapterRemovalV2 was used to perform Phred quality score-based screening before the raw readings were preprocessed (6). The *de novo* assembly was performed using the Unicycler assembler v0.4.8 (7). Genome annotation was performed by PGAP v5.2 (8), and antibiotic resistance genes were determined using AMRFinderPlus (9). Default parameters were used for all software. The taxonomic position of the strain was determined by Multilocus Sequence Typing v2.0 (10) and serotype by SerotypeFinder 2.0 (11). The total length of the draft genome of *Escherichia coli* was 4,931,994 bp containing 153 contigs with an N50 value of 151,408 bp and showed an average GC content of 50.6%. The genome coverage of the sample was identified to be 123×. The whole-genome sequencing revealed the isolated *Escherichia coli* strain belonged to the serotype O51:H6 and the sequence type ST2083 (12). Genome sequencing analysis revealed the presence of several antibiotic resistance genes which includes *gyrA(D87N*), *gyrA(S83L*), *parC(S80I), parE(S458A*), *aac(6')-Ib-cr5*, *drfA17, sul2 and tetB, bla*_NDM-5_, and *bla*_CMY-42_ predicted using AMRFinderPlus (9). In addition to resistance genes, virulence genes such as *anr*, *csgA*, *fdeC*, *fimH*, *gad*, *hlyE*,*lpfA*, *nlpI*, *terC*, *traT*, *yehB*, *yehC*, *yehD*, and *yfcV* were found using VirulenceFinder (13). Certain IS elements are also found in the genome CREC-1 belonging to the families IS630, IS4, IS3, ISAs1, IS66, ISL3, and IS5 identified using ISFinder(14). The Inc group of plasmids, IncFIB (AP001918), IncFII (*pAMa1167-NDM-5*), IncI(Gamma), IncX3, and IncX4, are an important class of plasmids identified using PlasmidFinder, found in *Escherichia coli* providing multidrug resistance (15).

## Data Availability

The draft genome sequence of *Escherichia coli* ST2083 has been deposited at DDBJ/ENA/GenBank under BioProject ID PRJNA754036 and BioSample id SAMN20721398 with the accession number JAIGYR00000000. The raw sequence data are available at NCBI SRA database SRR17245329.
